# Predicting the need for urgent endoscopic intervention in lower gastrointestinal bleeding: a retrospective review

**DOI:** 10.1186/s12873-024-00990-3

**Published:** 2024-04-23

**Authors:** Barzany Ridha, Nigel Hey, Lauren Ritchie, Ryan Toews, Zachary Turcotte, Brad Jamison

**Affiliations:** Department of Emergency Medicine, College of Medicine, University of Saskatchewan, Royal University Hospital, 104 Hospital Drive, Saskatoon, SK S7N 0W8 Canada

**Keywords:** Lower gastrointestinal bleeding, Emergency department, Endoscopy, LGIB

## Abstract

**Background:**

Lower gastrointestinal bleeding (LGIB) is a common reason for emergency department visits and subsequent hospitalizations. Recent data suggests that low-risk patients may be safely evaluated as an outpatient. Recommendations for healthcare systems to identify low-risk patients who can be safely discharged with timely outpatient follow-up have yet to be established. The primary objective of this study was to determine the role of patient predictors for the patients with LGIB to receive urgent endoscopic intervention.

**Methods:**

A retrospective chart review was performed on 142 patients. Data was collected on patient demographics, clinical features, comorbidities, medications, hemodynamic parameters, laboratory values, and diagnostic imaging. Logistic regression analysis, independent samples t-testing, Mann Whitney U testing for non-parametric data, and univariate analysis of categorical variables by Chi square test was performed to determine relationships within the data.

**Results:**

On logistic regression analysis, A hemoglobin drop of > 20 g/L was the only variable that predicted endoscopic intervention (*p* = 0.030). Tachycardia, hypotension, or presence of anticoagulation were not significantly associated with endoscopic intervention (*p* > 0.05).

**Conclusions:**

A hemoglobin drop of > 20 g/L was the only patient parameter that predicted the need for urgent endoscopic intervention in the emergency department.

**Supplementary Information:**

The online version contains supplementary material available at 10.1186/s12873-024-00990-3.

## Introduction

Lower gastrointestinal bleeding (LGIB) is a frequent cause of emergency department visits and subsequent hospitalizations with an estimated annual incidence of 20–30 per 100,000 people [1]. The differential for LGIBs is wide, and not all conditions require emergent or acute follow-up. Ideally, patients who are low risk may be observed as outpatients to minimize hospitalization time, iatrogenic harm, and healthcare costs [2]. Risk stratification strategies such as the Oakland Score and the Birmingham Score may be employed to inform these healthcare decisions [2, 3]. However, when acute intervention is needed, physicians may employ therapeutic endoscopy for diagnosis and treatment. While diagnostic algorithms exist for the use of endoscopy, predictive tools to determine which patients are most likely to receive it do not [2, 4].

Stratifying patients by baseline risk factors may be an effective way to triage LGIB and ensure patients receive optimized care [1, 2, 4]. While the current literature identifies variables which predict safe outpatient follow-up, such as lack of anticoagulation, sex, age, rectal examination findings, previous LGIB, systolic blood pressure, heart rate, and hemoglobin level [4], these same variables may not equate to which patients also require endoscopic intervention. Therefore, it is important to identify the overlap in effect, or lack thereof, between baseline patient characteristics on safe outpatient follow-up, and those which indicate endoscopic intervention.

## Methods

### Study design and time period

A retrospective cohort study of 142 patients conducted in the emergency departments at Regina General Hospital and Pasqua Hospital in Regina, Saskatchewan between October 2015, and October 2021.

### Study setting

The study was conducted within the emergency department of Regina General and Pasqua Hospitals in Regina, Saskatchewan, Canada.

### Population

The study population included male and female participants aged 18–99 years old who presented to the emergency department with chief complaint and primary diagnosis of rectal bleeding. Patients were identified with evidence of lower gastrointestinal bleeding (LGIB) based on International Classification of Diseases, (ICD-10) codes. Exclusion criteria included incomplete baseline values, pregnancy, and signs of upper GI source.

### Intervention

Primary intervention was urgent evaluation and management of LGIB by endoscopy.

### Outcome measures

The primary outcome measure was to retrospectively explore the role of patient factors and markers in the prediction of urgent endoscopic intervention need in the emergency department in patients with LGIB. The study aimed to identify patient predictors for intervention, through evaluation of vital signs, baseline lab values, and initial bloodwork on presentation to the emergency department.

### Data analysis

The variables assessed within the study were triage heart rate, triage blood pressure, history of colorectal cancer, history of anticoagulation use, initial hemoglobin and hemoglobin drop from baseline, INR, time in emergency department, and endoscopic report requiring intervention (Y/N), some of which comprising of the data points within the Oakland and Birmingham scores [2, 3]. Associations were compared with logistic regression testing. Tachycardia was defined as a heart rate of ≥ 90 bpm [5]. Univariate analysis of categorical variables by Chi square test was performed. A t-test was performed to determine if hospital length of stay differed in patients with a hemoglobin loss of ≥ 20 g/L. All statistical analysis was performed using SPSS (IBM SPSS Statistics 26.0).

### Sample size

**Fig. 1:**
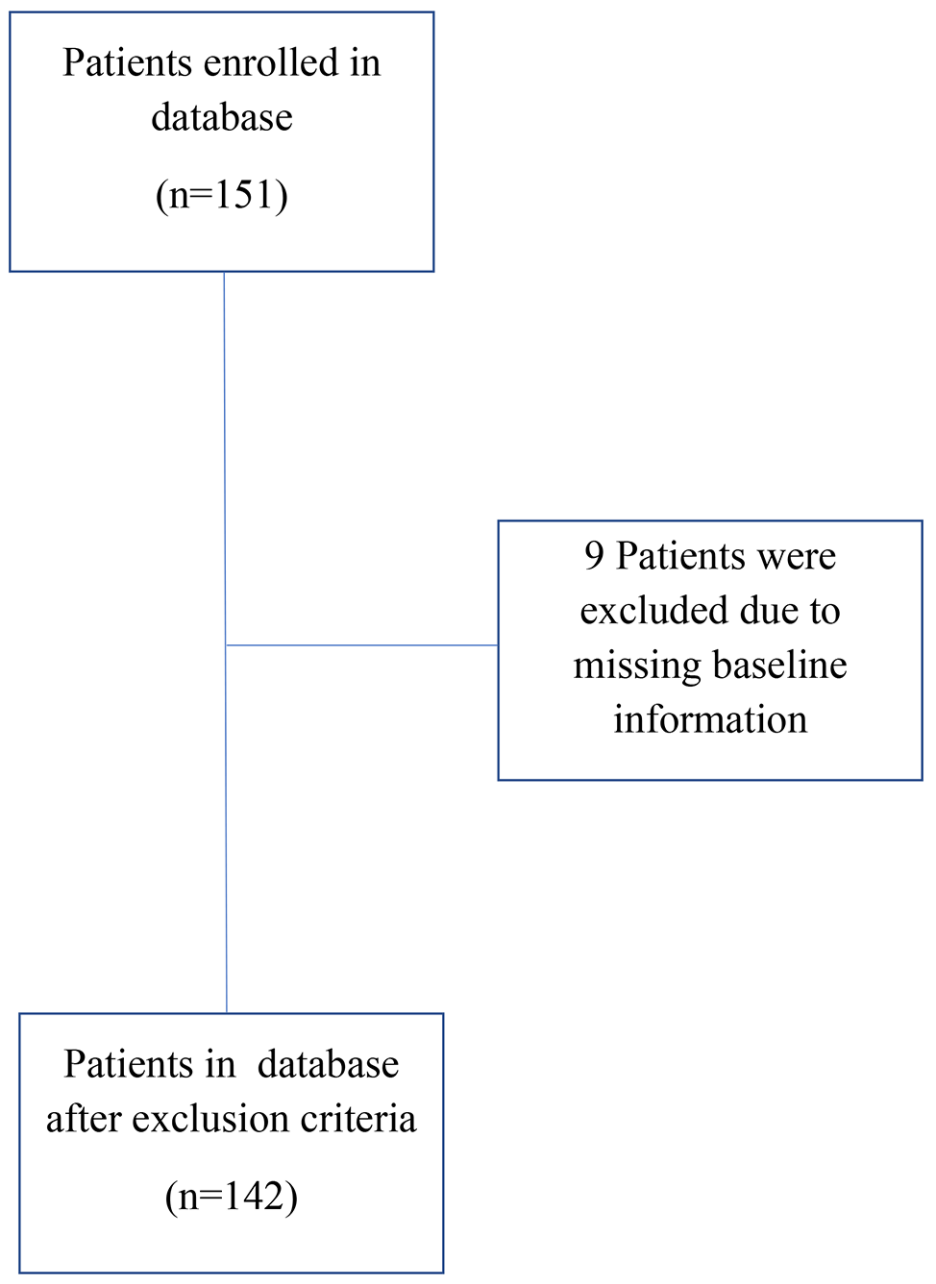
Patient enrolment flowchart

A convenience sampling method was used for the recruitment of study participants, with a total of 142 patients included in the study. See Fig. 1.

### Ethics board

The research ethics board that approved this research was the Saskatchewan Health Authority Research Ethics Board. Ethics certificate number OA-SHA-21-71.

## Results

142 patients were included and the distribution of their data can be seen in Table 1. 9 were excluded due to incomplete data (Fig. 1). At triage, 45.8% were tachycardic, with an average heart rate of 90.2 (σ = 20). Average Mean Arterial Pressure (MAP) was 47.5 mmHg (σ = 8.1) and 3.5% were hypotensive. Anticoagulation was used by 16.2% of patients, with 6.3% using warfarin, 4.2% on rivaroxaban, 0.7% on dabigatran, 0.7% on enoxaparin, 3.5% on apixaban, and 0.7% on heparin. The average hemoglobin drop was 18.47 g/L (σ = 18.8 g/L) and a hemoglobin drop of > 20 g/L was observed in 36.1% of patients. All patients underwent colonoscopy, and 46.1% underwent an intervention during colonoscopy. Average length of stay in the ED was approximately 17 h. The significance level was set at *P* < 0.05.

**Table 1:** Demographic, Medication, and Intervention Statistics

Demographics	
Sample size	142
**Variables**	
Tachycardia at triage	65 (45.8%)
Systolic hypotension at triage	5 (3.5%)
Hgb drop > 20	48 (36.1%)
Hemoglobin data not available	9 (12.8%)
On anticoagulant	23 (16.2%)
Anticoagulant by type:	
Warfarin	9 (6.3%)
Rivaroxaban	6 (4.2%)
Apixaban	5 (3.5%)
Dabigatran	1 (0.7%)
Enoxaparin	1 (0.7%)
Heparin	1 (0.7%)
**Intervention**	
Received colonoscopy	142 (100%)
Underwent intervention during colonoscopy	65
Underwent intervention for hemostasis during colonoscopy	16
Hemostatic Clipping	14
Polypectomy	25
Endoscopic band ligation	1
Argon Plasma Coagulation	1
Biopsy	17

Regression analysis performed on all clinical characteristics demonstrated a significant relationship only between a hemoglobin drop of > 20 and endoscopic intervention (Table 2). Chi square analysis suggested a strong relationship between a hemoglobin drop > 20 g/L, and endoscopic intervention (χ2 = 5.884, *p* = 0.015, df = 1) (Tables 3 and 4). Tachycardia, hypotension, or presence of anticoagulation were not significantly associated with endoscopic intervention with *p* = 0.272, *p* = 0.278, and *p* = 0.405.

**Table 2:** Variables predictive of intervention

	B	S.E.	Wald	df	Sig	Exp(B)
Systolic hypotension	1.250	1.152	1.178	1	0.278	3.490
Tachycardia	− 0.398	0.363	1.206	1	0.272	0.671
Hgb drop > 20	0.826	0.380	4.728	1	**0.030**	2.285
Constant	− 0.337	0.278	1.472	1	0.225	0.714

**Table 3:** Chi-square analysis of systolic hypotension, tachycardia, and hemoglobin drop > 20

	Chi-square	df	Sig.
Systolic hypotension, tachycardia, Hgb drop > 20	8.587	3	**0.035**

**Table 4:** Chi-square analysis of hemoglobin drop > 20 as single variable

	Chi-square	df	Sig.
Hgb drop > 20	5.884	1	**0.015**

Comparison between patients who suffered a hemoglobin drop of ≥ 20 g/L versus those who did not was performed using t-testing. Length of stay in hospital was not significant between both groups.

## Discussion

### Interpretation of findings

Our data suggests a hemoglobin drop of 20 points was predictive of intervention. When triaging patients for endoscopic evaluation of LGIB, hemoglobin concentrations may inform clinicians as to the severity of the bleed, and circumstantial acuity. This research also suggests LGIB management should begin with an assessment of patient hemoglobin both for consideration of endoscopic evaluation or safe patient discharge.

### Comparison to previous studies

Existing literature attempts to characterize which patient factors merit intervention in LGIB. While LGIB alone account for almost 1% of ED admissions [6], widely accepted criterion for endoscopic intervention do not exist [6]. Furthermore, a minority of LGIB deserve endoscopy. In a study by Ng et al., only 10% of LGIB patients required endoscopic intervention [7]. Similarly, a significant cohort of LGIB patients may be safely discharged without the need for intervention. Patel et al. described three validated parameters which predicted criteria for safe discharge of LGIB patients; hemoglobin > 13 g/dL, systolic BP > 115mmHg, and no anticoagulation [8]. One scoring tool, the Oakland criteria, aggregates age, male sex, prior GI bleed, digital rectal exam (DRE) findings, elevated heart rate, elevated blood pressure, and low hemoglobin to determine the acceptability of outpatient management of LGIB [9]. A systematic review by Almaghrabi et al. suggested the Oakland criteria was most accurate in predicting safe discharge, need for non-endoscopic intervention, and was the most effective triage tool for LGIB [10].

The need for intervention of any kind increases with the extent of hemoglobin loss [7, 8]. However, our research does not correspond with variables commonly cited in the literature as being known to reduce the need for intervention or even support discharge for outpatient management (1,2,4,11). Although there is ample data on the safety of early discharge, there is a notable scarcity of information on the need for endoscopic intervention. Predictive variables for discharge and outpatient management do not necessarily indicate predictability for the need of endoscopy.

### Strengths and limitations

Given the paucity of data available for informing guidelines around endoscopic intervention, this research is unique. Also, our key findings were in keeping with those of larger studies mentioned. This study also elucidates an important question, in that the indications for when endoscopy may be necessary, may differ from those which are suggestive of prioritizing supportive management in isolation.

Still, certain limitations should be considered when interpreting the results. Firstly, the study size population was only comprised of 142 participants, which may detriment the statistical power of the analysis. Finally, a lack of follow-up in patients who were discharged may have limited the ability to fully evaluate the long-term outcomes in discharge, or the need for endoscopic intervention after discharge. Further research may reveal the ideal praxis around early discharge and endoscopic intervention in this population.

### Clinical implications

This research serves to benefit clinicians in multiple ways. In the initial workup and management of a patient with a LGIB, clinicians should use the hemoglobin as an important gauge for severity, and the subsequent consideration of endoscopic management. Also, this research suggests that certain variables may be less important when evaluating the need for endoscopy. Early identification of patients who may require endoscopic management may reduce the need for unnecessary transfusion, failure of supportive care, and invasive treatment. Contrarily, triaging patients and ideally reducing endoscopy utilization may optimize patient care, and reduce the need for unnecessary intervention.

### Research implications

At current, no algorithms or clinical pathways exist to assess the need for endoscopic intervention. While these research findings are agreeable to similar literature, it poses an important question; Is there a difference in variables which predict the need for endoscopy, as compared to which variables predict safe supportive treatment, and to what extent does an overlap exist between these two processes. It is important that the literature attempt to characterize the above with future research.

## Conclusion

In conclusion, this study highlights which patient variables predict the need for endoscopic intervention in patients with lower gastrointestinal bleeding (LGIB). The findings suggest that a hemoglobin drop of greater than 20 points is predictive of intervention, which could inform clinicians when triaging patients for endoscopic evaluation or safe patient discharge. While overlap with existing literature exists regarding which variables are predictive, the lack of contiguity with other variables identified in the research surrounding safe discharge and safe outpatient management of LGIB suggest that the variables which predict endoscopy may differ.

### Electronic supplementary material

Below is the link to the electronic supplementary material.

Supplementary Material 1

## Data Availability

Data is available for viewing upon reasonable request from the corresponding author after consultation with our local Ethics board.
