# Supplementary material for: Predicting the need for urgent endoscopic intervention in lower gastrointestinal bleeding: a retrospective review
Source: BMC Emerg Med. 2024 Apr 23;24:71. doi: 10.1186/s12873-024-00990-3 (PMC11040937; doi:10.1186/s12873-024-00990-3)
Supplement: Supplementary file 1 — Supplementary Material 1 [file 12873_2024_990_MOESM1_ESM.docx]

**Clinicians Capsule**

**What is known about the topics?**

Lower gastrointestinal bleeding (LGIB) is a common reason for emergency department visits, however patient predictors indicating urgent endoscopic intervention remain largely unknown.

**What did I ask?**

Are there predictors in patients with LGIB that can predict the need for urgent endoscopic intervention.

**What did this study find?**

A 20-point drop in hemoglobin predicted need for urgent endoscopic intervention.

**Why does this study matter to clinicians?**

This study can guide clinicians in identifying whether patients with LGIB need urgent endoscopic intervention, and minimize delays to treatment.
